# Got Milk? Breastfeeding and Milk Analysis of a Mother on Chronic Hemodialysis

**DOI:** 10.1371/journal.pone.0143340

**Published:** 2015-11-16

**Authors:** Michael S. Balzer, Mechthild M. Gross, Ralf Lichtinghagen, Hermann Haller, Roland Schmitt

**Affiliations:** 1 Department of Nephrology and Hypertension, Hannover Medical School, Hannover, Germany; 2 Midwifery Research and Education Unit, Hannover Medical School, Hannover, Germany; 3 Institute of Clinical Chemistry, Hannover Medical School, Hannover, Germany; Emory University, UNITED STATES

## Abstract

**Purpose:**

Women on dialysis rarely become pregnant. However, the overall rate of successful pregnancies is increasing in this patient population and breastfeeding becomes an option for mothers on dialysis. In this study we performed a systematic breast milk composition analysis of a mother on chronic hemodialysis (HD).

**Methods:**

Specimens of breast milk and blood were collected in regular intervals before and after HD from a 39-year old woman starting on day 10 postpartum. Samples were analyzed for electrolytes, retention solutes, nutrients and other laboratory measurements. Breast milk samples from low-risk mothers matched for postpartum age were used as controls.

**Results:**

Significantly higher levels of creatinine and urea were found in pre-HD breast milk when compared to post-HD. A similar post-dialytic decrease was only found for uric acid but not for any other investigated parameter. Conversely, sodium and chloride were significantly increased in post-HD samples. Compared to controls creatinine and urea were significantly higher in pre-HD samples while the difference remained only significant for post-HD creatinine. Phosphate was significantly lower in pre- and post-HD breast milk when compared to controls, whereas calcium showed no significant differences. In terms of nutrient components glucose levels showed a strong trend for a decrease, whereas protein, triglycerides and cholesterol did not differ. Similarly, no significant differences were found in iron, potassium and magnesium content.

**Conclusion:**

To the best of our knowledge this is the first report on a breastfeeding mother on chronic dialysis. Although we found differences in creatinine, urea, sodium, chloride and phosphate, our general analysis showed high similarity of our patient’s breast milk to samples from low-risk control mothers. Significant variations in breast milk composition between pre- and post-HD samples suggest that breastfeeding might be preferably performed after dialysis treatment. In summary, our findings indicate that breastfeeding can be considered a viable option for newborns of mothers on dialysis.

## Introduction

Due to endocrine abnormalities and sexual dysfunction, fertility of chronic kidney disease (CKD) and end stage renal disease (ESRD) patients of childbearing age is generally reduced[1]. Accordingly, the incidence of pregnancies in women on chronic dialysis is very low but appears to be increasing from 0.9% in 1980[2] to about 1.0–7.0% in the 1990s[3–7]; still, the course of pregnancy remains challenging for both mother and child[8]. With intensified hemodialysis (HD) regimens[9], however, the prevalence of maternal complications and adverse fetal outcomes has decreased encouragingly and more term infants are born[10–13]. Overall rates of successful pregnancies, i.e. resulting in a live infant, reach up to 71–87%[11, 14], gestational age has increased considerably and maternal complications have decreased dramatically within the last few decades[11, 15, 16].

In a 2012 statement the American Academy of Pediatrics reaffirmed its recommendations of breastfeeding as normative standard for newborn and infant feeding due to beneficial short- and long-term effects[17]. Advantages of breastfeeding include developmental[18], economic[19, 20], health, nutritional, immunological, psychological, social and environmental benefits[17]. Recently, a systematic review of the long-term effects of breastfeeding by the World Health Organization concluded that breastfeeding might decrease obesity risk during childhood and adolescence and that there is strong evidence of a causal relationship to intelligence quotient[21–23]. Others could show that breastfeeding reduces the risk of developing diabetes type 2 and several additional cardiovascular risk factors[24]. Mother-infant separation may be common in women after having experienced a complicated pregnancy or childbirth. This is limiting the beneficial aspects of breastfeeding and early skin-to-skin contact (SSC) for the newborn. SSC does not only have immediate effects on basic biological functions, such as blood glucose levels, but has also been recognized as an essential element of the newborn period for programming physiology and behavior in the infant[25, 26].

Data on breastfeeding mothers on chronic dialysis are lacking and to the best of our knowledge, there are no studies that have analyzed breast milk and its components in women with CKD. In this study we analyzed breast milk of a mother on chronic HD in a longitudinal fashion and compared milk composition to breast milk of low-risk control mothers.

## Materials and Methods

### Subjects and sample collection

Starting on day 10 postpartum, regular specimens of breast milk were collected, frozen at -80°C and analyzed at a later time point. Samples were collected until week 10 postpartum when the mother on HD decided to wean the infant. For controls, breast milk specimens of healthy mothers (n = 6) of about the same age (34 ± 3.10 years) without any history of renal disease or any other serious medical conditions were collected at corresponding postpartum time points and analyzed later. The patient and all control subjects gave written informed consent to participate in the study, which had been approved by the institutional ethics board of Hannover Medical School.

### Dialysis regimen

HD treatments were performed as outlined in the results section and standardized to a blood and dialysate flow of 300 mL/min (Qb) and 500 mL/min (Qd), respectively. HD was performed with high-flux polysulfone filters using individualized bicarbonate levels ranging from 26–30 mmol/L. Dialysate sodium and bicarbonate were set to 136.0 mmol/L and 26.0 mmol/L, respectively, and remained constant throughout the entire study period. No sodium profiling was used. Dialysate chloride was standardized to 110.5 mmol/L, dialysate calcium to 1.25 mmol/L, dialysate magnesium to 1.0 mmol/L and dialysate acetate to 3.0 mmol/L. Dialysate potassium concentration at 3.0 mmol/L was used continuously throughout the study period. Weekly standard Kt/V (urea) (stdKt/V) was calculated on a regular basis (every 3 months) from number of treatments per week, duration of treatment, pre- and post-dialysis weight and urea concentrations.

### Biochemical analysis

All measurements were performed on a *cobas 6000 analyser system* (*Roche Diagnostics*, Mannheim, Germany). Sodium, potassium and chloride were measured potentiometrically on the ISE module. The other biochemical parameters, including retention solutes such as creatinine, urea and uric acid, were measured photometrically as follows: creatinine (CREP2; enzymatic colorimetric method), urea (UREAL; kinetic urease test with GLDH), phosphate (PHOS2; molybdate UV method), uric acid (UA2; uricase enzymatic colorimetric test), LDH (LDHI2; enzymatic UV assay), magnesium (MG2; xylidyl blue colorimetric endpoint method), total protein (TP2; colorimetric biuret assay), albumin (ALBT2; immunoturbidimetric assay), glucose (GLUC2; enzymatic hexokinase method), triglycerides (TRIGB; enzymatic colorimetric assay), cholesterol (CHOL2; enzymatic colorimetric method), iron (IRON2; colorimetric assay), calcium (CA2; NM BAPTA method), respectively. Immunoglobulins A, G, M and E were measured with immunoturbidimetric assays (Tina-quant Gen.2, Roche Diagnostics, Mannheim, Germany) on a cobas c501 system.

### Statistical analysis

Statistical analysis was performed using a two-tailed paired t test for longitudinal pre-post data. Comparisons with controls were analyzed using a two-tailed unpaired t test, data was computed with GraphPad Prism and Microsoft Excel. Results are given as means with standard error of the mean (SEM), unless otherwise stated. Changes were considered statistically significant for p < 0.05 (two-sided).

## Results

**Fig 1:**
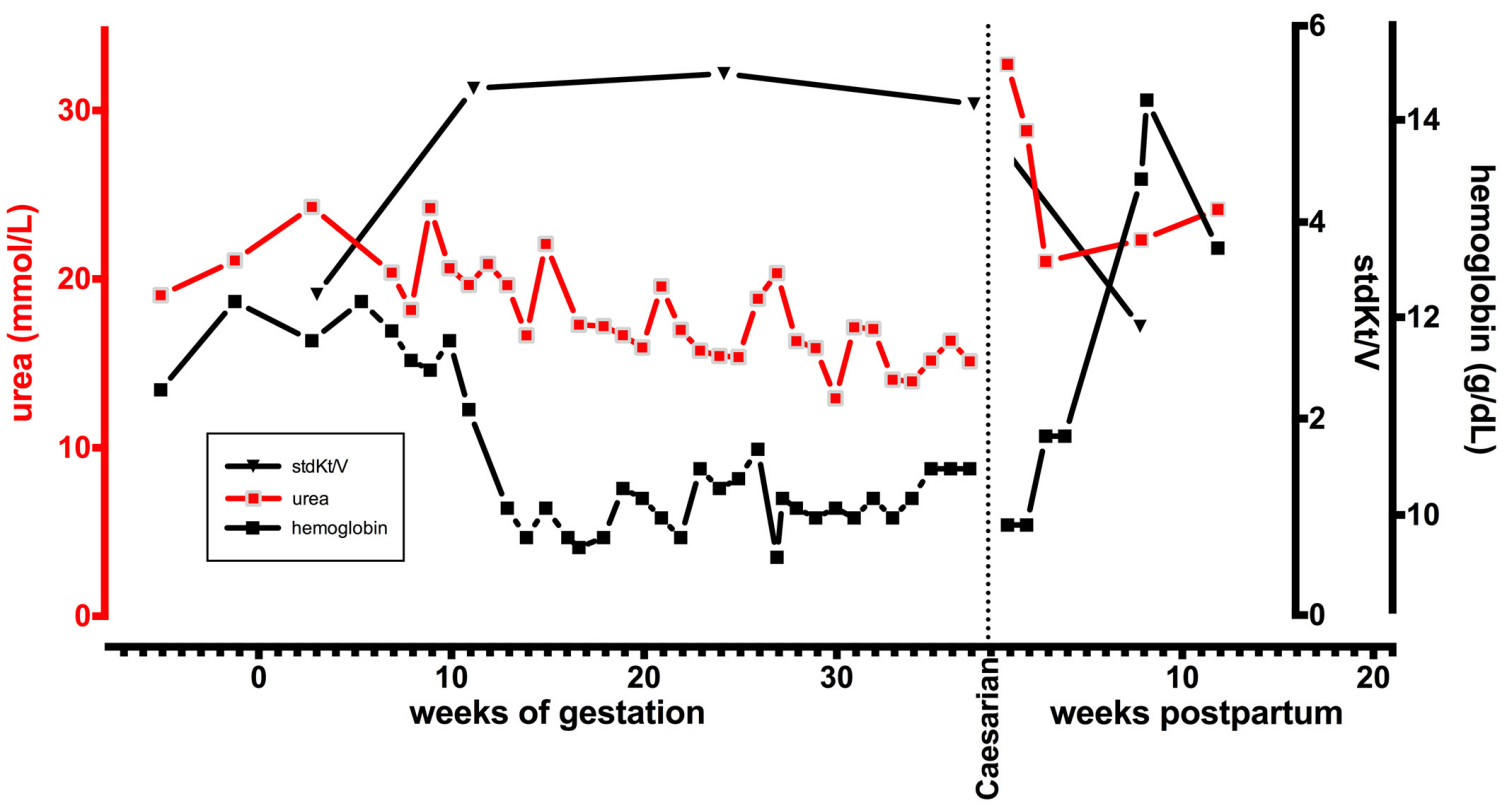
Longitudinal development of urea, hemoglobin and stdKt/V during gestation and postpartum. Note the increase of stdKt/V with intensified HD regimen during pregnancy.

A 39-year-old caucasian secundipara (third gravida) after spontaneous birth with a 5 ½ year history of ESRD secondary to hypertensive nephrosclerosis was confirmed to be 7 weeks pregnant. At this point her HD regimen was intensified from 3 times per week 8 hours to 6 times per week 5 hours. Her stdKt/V rose accordingly from 3.23 ± 0.05 per week during the 12 months prior to pregnancy to 5.37 ± 0.16 during pregnancy. Pregnancy was uneventful and fetal growth and development were appropriate for gestational age. At 37 6/7 weeks of gestation a healthy male baby was delivered by elective Caesarian section (3090 grams, 54 cm, Apgar score of 9–10). After delivery, HD regimen was switched to standard 5 hours 3 times a week and the mother stated her strong wish to breastfeed the infant. Longitudinal development of urea, hemoglobin and stdKt/V during gestation and postpartum is depicted in Fig 1. The mother’s serum labwork is summarized in Table 1.

**Table 1:** Serum labwork.

sodium (mmol/L)	136.00 ± 1.22
potassium (mmol/L)	5.92 ± 0.45
chloride (mmol/L)	105.26 ± 2.11
creatinine (μmol/L)	663.00 ± 31.82
urea (mmol/L)	25.54 ± 4.85
uric acid (μmol/L)	442.53 ± 64.83
calcium (mmol/L)	2.14 ± 0.04
phosphate (mmol/L)	2.29 ± 0.17
iPTH (ng/L)	165.80 ± 18.10
25OH-vitamin D3 (μg/L)	21.93
glucose (mmol/L)	4.98 ± 2.63
protein (g/dL)	6.50 ± 0.37
albumin (g/L)	42.30
triglycerides (mmol/L)	1.16
cholesterol total (mmol/L)	5.13
HDL cholesterol (mmol/L)	1.18
LDL cholesterol (mmol/L)	3.58
hemoglobin (g/dL)	11.99 ± 1.85
hematocrit (%)	36.81 ± 5.77
thrombocytes (/nL)	256.13 ± 66.16
CRP (mg/L)	5.43 ± 6.92

Data are presented as mean ± SD where appropriate or as single values, respectively, if only measured once during the lactation period. iPTH, intact parathyroid hormone; HDL, high density lipoprotein; LDL, low density lipoprotein; CRP, C-reactive protein.

**Fig 2:**
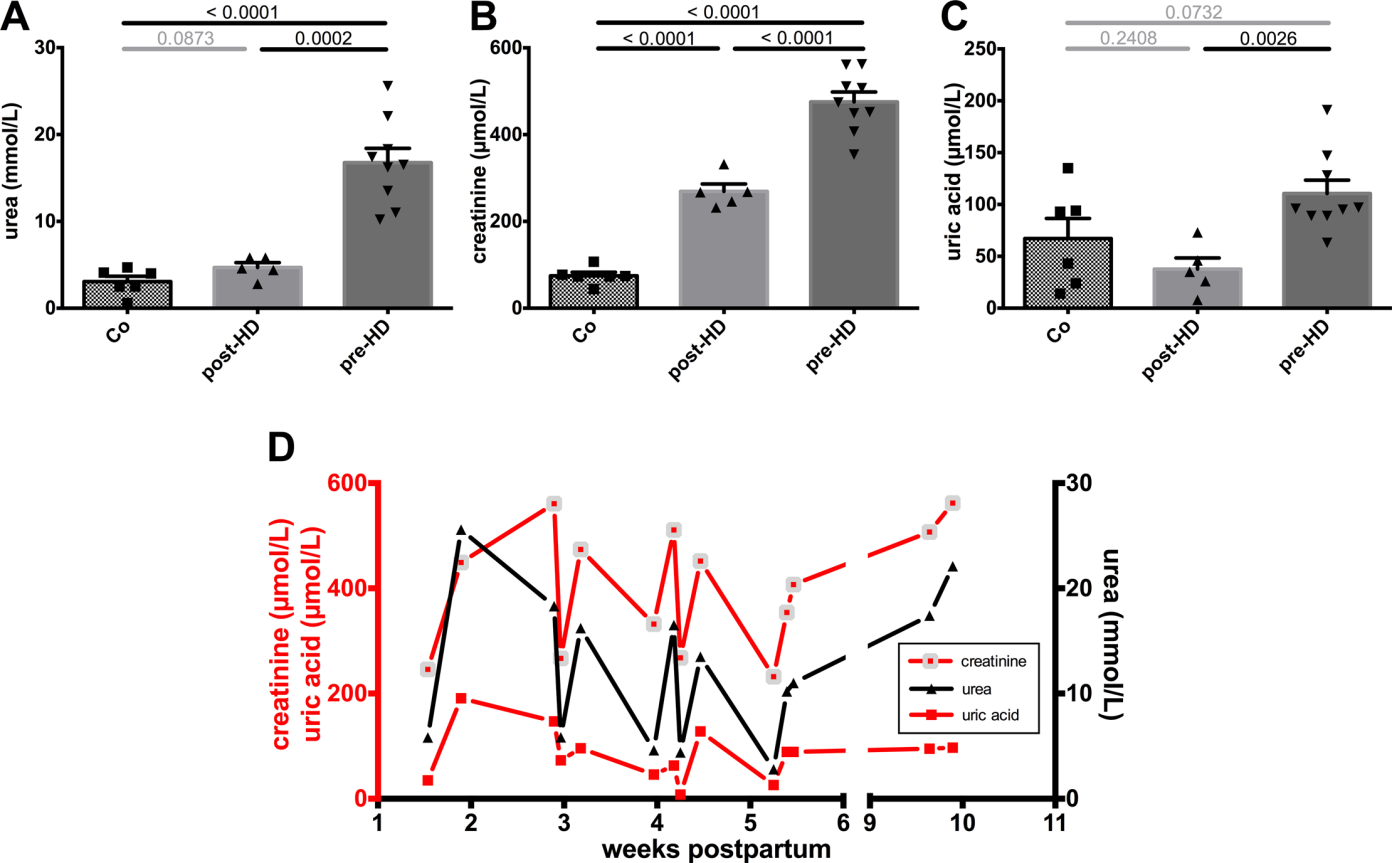
Retention solutes concentrations in breast milk. **A, B.** Breast milk retention solutes of our mother on dialysis were significantly elevated prior to HD (pre-HD) when compared to healthy controls (Co), as shown for urea and creatinine. HD treatment significantly reduced breast milk creatinine concentration and even normalized urea to control level (post-HD). **C.** Similarly, pre-HD elevated uric acid concentrations normalized post-HD. **D.** A strong relationship of breast milk retention solute concentration with HD treatments could be seen and is visualized in a longitudinal fashion.

A pragmatic nutritional approach including a combination of breast milk and formula feeding was chosen. Repetitive specimens of breast milk were collected to be analyzed for uremic metabolites, electrolytes, nutritional components and other parameters. Breast milk samples from before and after HD sessions were compared to control samples from low-risk control mothers who were matched for postpartum age. Analytical breast milk labwork from the mother and controls is presented in Table 2. Urea and creatinine concentrations were significantly higher in pre-HD samples when compared to post-HD samples (Fig 2A and 2B). Post-HD samples were still significantly higher in creatinine but not in urea when compared to control samples (Fig 2A and 2B). Uric acid also showed a significant decrease between pre- and post-HD breast milk and the post-HD concentration was similar to control samples (Fig 2C). The strong relationship of breast milk retention solute concentrations prior to and post HD with HD treatments is depicted in Fig 2D.

**Table 2:** Breast milk labwork.

	mother on HD	controls (n = 6)
sodium (mmol/L)	18.79 ± 8.60	9.83 ± 1.72
chloride (mmol/L)	17.50 ± 6.48	9.50 ± 1.38
potassium (mmol/L)	16.16 ± 2.47	15.00 ± 2.73
magnesium (mmol/L)	1.55 ± 0.18	1.40 ± 0.28
calcium (mmol/L)	7.69 ± 0.77	6.88 ± 1.03
phosphate (mmol/L)	0.81 ± 0.36	1.86 ± 0.56
creatinine (μmol/L)	401.57 ± 117.78	74.67 ± 19.99
urea (mmol/L)	12.44 ± 7.19	3.07 ± 1.51
uric acid (μmol/L)	84.50 ± 49.13	67.17 ± 47.46
glucose (mmol/L)	0.84 ± 0.31	1.51 ± 0.63
total protein (g/L)	11.39 ± 5.48	7.93 ± 4.94
albumin (g/L)	0.42 ± 0.06	0.45 ± 0.05
triglycerides (mmol/L)	29.21 ± 14.69	26.22 ± 23.32
cholesterol (mmol/L)	0.41 ± 0.18	0.36 ± 0.27
iron (μmol/L)	17.74 ± 16.01	19.04 ± 11.47
LDH (U/L)	465.07 ± 185.55	346.50 ± 162.01
GGT (U/L)	1411.43 ± 516.88	1712.33 ± 551.05
amylase (U/L)	3345.25 ± 778.55	1911.80 ± 725.21
lipase (U/L)	1723.64 ± 470.91	1163.83 ± 533.67

Data are presented as mean ± SD. LDH, lactate dehydrogenase; GGT, gamma glutamyl transferase.

**Fig 3:**
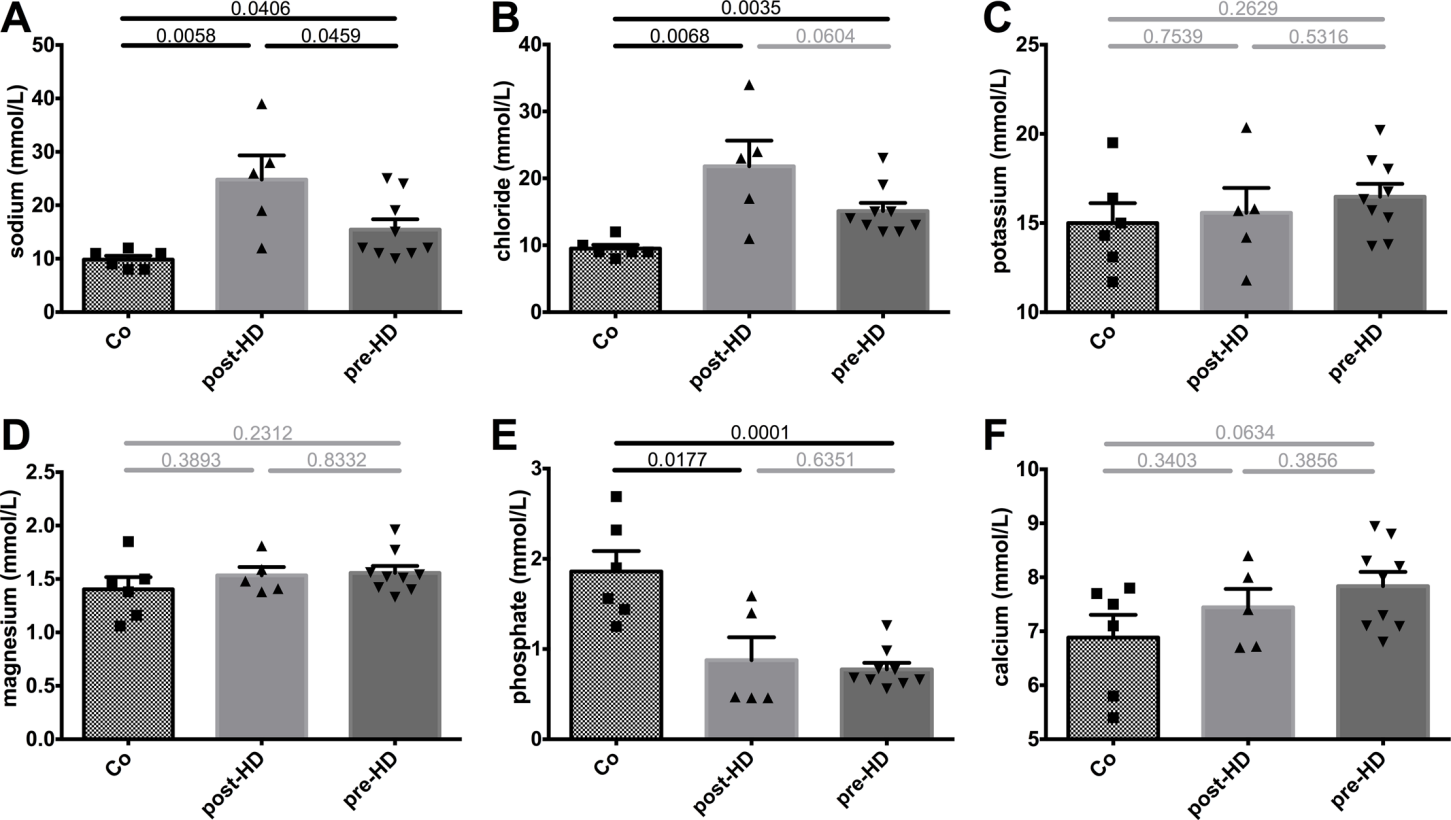
Electrolyte and phosphate concentrations in breast milk. **A-D.** Electrolyte composition of breast milk showed only minor changes pre- and post-HD, but HD treatment lead to a significant increase of sodium and chloride concentrations of breast milk when compared to Co, which–in the case of sodium–was still present prior to HD. **E-F.** Phosphate levels were similar pre- and post-HD but were significantly lower than in Co, whereas calcium levels did not differ significantly.

Conversely, sodium, chloride, potassium and magnesium showed only minor changes between pre- and post-HD but sodium and chloride concentrations were significantly higher when compared to control samples (Fig 3A–3D). Interestingly, phosphate levels were similar between pre- and post-HD but were significantly lower when compared to healthy controls (Fig 3E) despite elevated serum phosphate levels with a mean of 2.29 ± 0.17 mmol/L during the observation period. Calcium levels did not differ significantly (Fig 3F).

**Fig 4:**
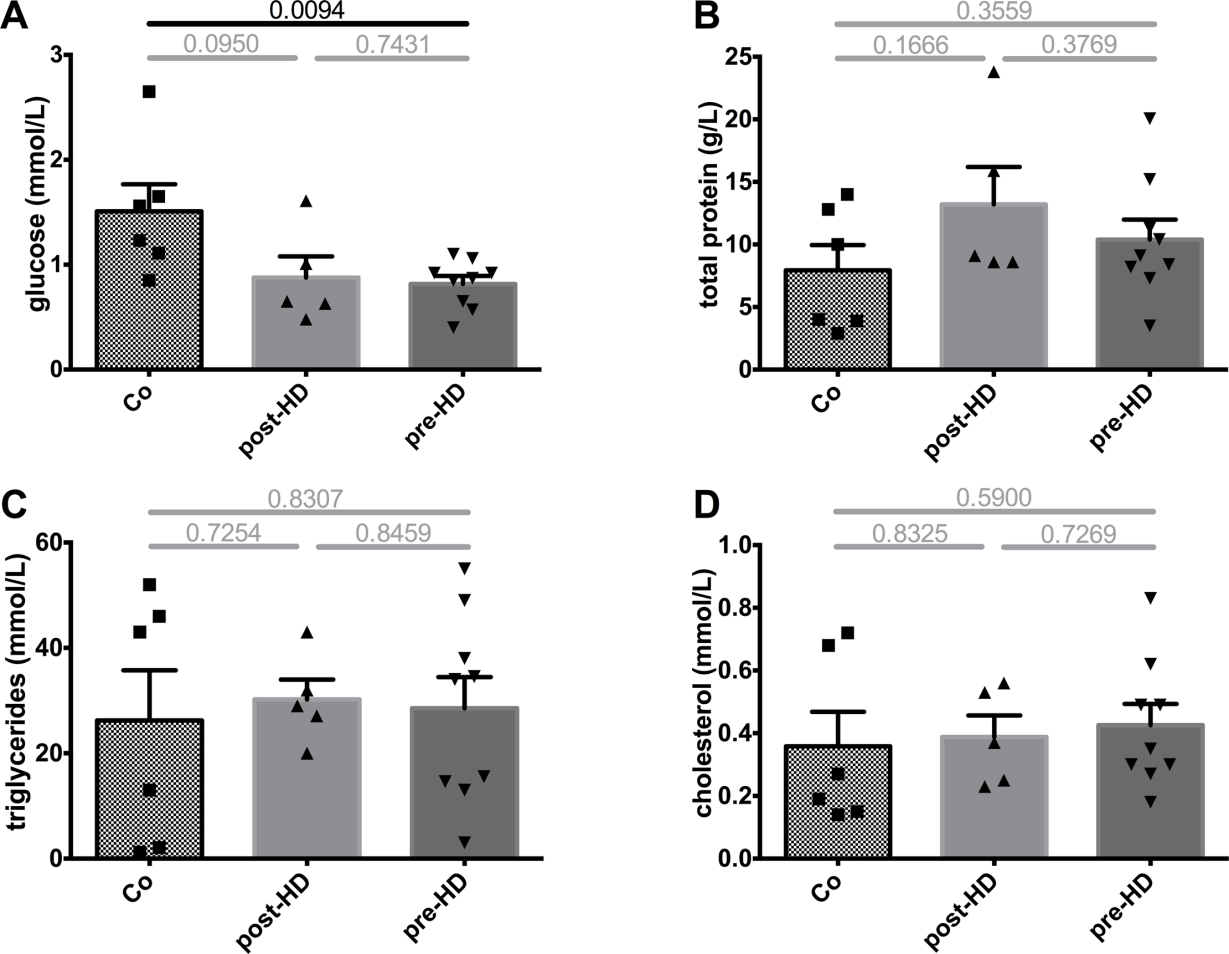
Nutrient components in breast milk. **A.** Compared to Co, breast milk glucose levels were generally lower in our patient. HD did not influence post-HD levels compared to pre-HD levels. **B-D.** In contrast, total protein, triglycerides and cholesterol did not differ significantly when compared to controls. Again, HD did not influence post-HD levels compared to pre-HD levels.

In terms of nutrient components, breast milk glucose levels were unchanged between pre- and post-HD samples but were generally lower when compared to control samples (Fig 4A). Concentrations of total protein, triglycerides and cholesterol did not differ between pre- or post-HD or when compared to controls (Fig 4B–4D). Importantly, immunoglobulin levels of classes A, M and G did not differ from controls (data not shown, see S1 File), indicating that breast milk of our patient provided similar immunological protection as control milk. Of note, in all samples IgE concentrations were below the detection limit of 0.1 g/L.

## Discussion

Human milk is recommended as the exclusive nutrient source for term infants at least during the first six months of life[17]. It provides all the nutrients required for neonatal growth and seems to be associated with larger kidney volume and higher GFR in adulthood[27]. Breast milk has a variable composition that changes with several factors, such as postnatal age and maternal diet[28, 29]. During normal pregnancy and lactation renal function undergoes pronounced changes, which are altered or absent in ESRD patients[30, 31]. Although outcomes of pregnancies of mothers with CKD and ESRD have improved there are no guidelines on postpartum care in women with CKD and ESRD available.

In our study we found that the composition of breast milk from a woman undergoing maintenance dialysis differed from healthy control mothers in several aspects. While nutrient content was similar to control milk, mineral content and uremic substances showed significant changes according to the time of milk sampling before or after dialysis treatment. Analysis of pre- and post-dialysis samples revealed a drop in urea, creatinine and uric acid that paralleled the expected dialysis-dependent changes in blood levels. Consequently, post-dialysis milk samples were more similar to milk from control mothers than pre-dialysis samples. However, milk levels of creatinine were still significantly higher as well as levels of sodium and chloride, which showed a non-significant increase from pre- to post-dialysis. Given the substantial changes of concentrations in uremic substances we advised our patient to breastfeed preferentially after dialysis treatments while discarding milk that had been expressed towards the end of inter-dialytic intervals. A potential disadvantage of this recommendation could be the significant increase of breast milk sodium concentration we observed with hemodialysis treatments. Normally, breast milk sodium is initially high but declines as lactation is established and volumes increase. Persisting high breast milk sodium concentrations, as seen in our patient, can be indicative of lactation failure[32]. Sodium is actively secreted into milk, a process which is under tight hormonal control[33]. Hormonal alterations, potentially due to ultrafiltration-dependent volume changes, might have played a role in our patient. Indeed, it is known that sodium intake relates to increased blood pressure also in newborns and infants[34–36]. Beyond a finding that breastfeeding lowers blood pressure in later life of prematurely born children[37], it has been shown that an average reduction of around 50% of sodium intake in infants leads to a significant decrease in systolic blood pressure[38]. As it is known that, especially for infants, major sources of sodium are breast milk and infant formula[39], one could speculate that sodium loading of breast milk via hemodialysis would have negative implications on net sodium intake balance. However, as Maalouf and colleagues demonstrated that only 22.9% of total dietary sodium intake is due to human milk, whereas 71.7% is secondary to formula milk[39], and as sodium concentration of post-dialysis breast milk in our patient was only doubled when compared to controls, we estimate the cumulative dietary sodium intake of our patient’s newborn to be in the range of children fed with formula milk exclusively.

Another benefit of breastfeeding is the stimulatory effect of breast milk on neonatal gastrointestinal humoral immunologic development, namely IgA secretion, leading to a decrease in diarrhea incidence[40]. Also, there is longstanding evidence that maternal passive immunity decreases respiratory and other infections[41]. Interestingly, the general dialysis population has a 300 fold higher risk of sepsis than a control population matched for age, gender and diabetes prevalence[42], which is partly due to very poor antibody-mediated responses to infections. Similarly, vaccination response is generally significantly lower in dialysis patients[43]. We therefore evaluated breast milk concentrations of immunoglobulin subclasses IgA, IgG and IgM and were surprised to find no differences in comparison to controls. We therefore conclude that breastfeeding during HD is safe and that transmission of maternal passive immunity is probably not hampered.

Interestingly, phosphate levels were lower in our patient’s milk samples when compared to control milk despite elevated serum levels (average concentration during lactation period 2.29 ± 0.17 mmol/L). Phosphate concentrations were not influenced by dialysis treatments. Generally, postnatal growth and development require a considerable amount of phosphate and phosphate content is normally much higher in milk than in plasma[44]. Our patient showed the opposite pattern with an inversed milk to plasma ratio. In order to avoid nutritional deficiency of phosphate our patient used formula feeding containing a high phosphate concentration. The mechanism of phosphate transport by the lactating mammary epithelium is complex and includes phosphate transport across the basolateral membrane via a sodium phosphate cotransporter and phosphate secretion into milk via the Golgi vesicle route[44]. Our data indicate an altered mechanism of phosphate handling in our patient. One possible explanation could be that the sodium phosphate cotransporter was inhibited by changes in vitamin D and/or iPTH as described for renal and intestinal sodium phosphate cotransport[45]. In contrast to phosphate, milk of our patient showed a normal enrichment in calcium. Calcium levels were several folds higher than serum concentrations and similar to breast milk content in control mothers. The high amount of calcium which is excreted into milk during lactation is thought to be mobilized in the lactating mother via a temporary demineralization of the skeleton[46]. This process seems to be normally mediated by the release of PTH related peptide (PTHrP) from the lactating mammary gland[46]. In the context of CKD/ESRD and lactation there is no data available on potential changes in the regulation of PTHrP release which we did not determine in our patient. However, secondary hyperparathyroidism related to ESRD which was present in our patient (average iPTH levels during lactation period were 165.80 ± 18.10 pg/mL) can be expected to have interfered with the normal physiological signaling axis[46]. Although we do not provide any data on the severity of associated osteopenia of our patient, information about long-term bone health would be of interest and should be monitored in future patients.

The main weakness of our study lies in its casuistic character implying the problem of extrapolation from individual patient data. However, given that this is the first report on breast milk composition during chronic dialysis treatment, there are implications for further studies and potential reference points for postnatal care in ESRD. Most importantly, we found that the composition of our patient’s breast milk showed more similarities than discrepancies to milk from healthy mothers and we believe that mothers who wish to breastfeed despite CKD should be encouraged. One obstetrical limitation is the fact that elective Caesarean section was performed due to management challenges with HD. As this particular woman was a secundipara after spontaneous vaginal birth it is desirable to achieve further vaginal births in similar cases even in primiparity.

The quality of milk was affected in several aspects leading to several practical recommendations: 1. Elevated levels of uremic solutes can be avoided by preferentially feeding after dialysis treatment while expressing and discarding the milk towards the end of inter-dialytic intervals. 2. Formula milk should be used to bridge feeding pauses as it will also compensate for potential shortcomings in milk components, e.g. low phosphate content. 3. In order to protect the mother from excessive skeletal demineralization due to preexisting hyperparathyroidism together with lactation-driven calcium mobilization, calcitriol and vitamin D supplementation should be closely managed together with sufficient nutritional calcium intake. The recommendations refer to a healthy term born infant and would need to be modified in case of preterm birth or in children with inherited renal disease.

Without any doubts early SSC is beneficial to newborns for their immediate and later life[25, 26]. Women with chronic diseases, e.g. CKD, value the experience of pregnancy and motherhood as a healthy event[47]. They are usually very well informed about health risks, family burden and the perceived risk of fetal malformation. 'Fear of birth defects'[47] is one of the fears related to the potential side effects of immunosuppression. Women are determined to accept the risks of pregnancy while being able to enjoy a physiological pregnancy. Proactive counseling and shared decision-making offer woman-centered solutions. A multidisciplinary team involving nephrologists, obstetricians and midwives is required to ensure physiological labour and birth outcomes and immediate bonding during postpartum care.

## Supporting Information

S1 File Immunoglobulin concentrations in breast milk. Breast milk IgA, IgG and IgM concentrations did not differ between our patient and controls. In all samples IgE was below the detection level of 0.1 g/L.
(ZIP)
