# Supplementary material for: Got Milk? Breastfeeding and Milk Analysis of a Mother on Chronic Hemodialysis
Source: PLoS One. 2015 Nov 16;10(11):e0143340. doi: 10.1371/journal.pone.0143340 (PMC4646653; doi:10.1371/journal.pone.0143340)
Supplement: S1 File — Breast milk IgA, IgG and IgM concentrations did not differ between our patient and controls. In all samples IgE was below the detection level of 0.1 g/L. (ZIP) [file pone.0143340.s001.zip › 2015-10-21__SKMBT_2231815102116080.pdf]

Datenmonitor 21/10/15 16:12

Ser/Pl	N	00023-1			1
21/10/15	IGA-2		IGM-2	IGG-2	
15:28:40	1.458		0.486	0.22	
				<Test	
Ser/Pl	N	00023-2			2
21/10/15	IGA-2		IGM-2	IGG-2	
15:28:40	1.657		0.749	0.37	
				<Test	
Ser/Pl	N	00023-4			4
21/10/15	IGA-2		IGM-2	IGG-2	
15:28:40	2.096		0.474	0.22	
				<Test	
Ser/Pl	N	00023-5			5
21/10/15	IGA-2		IGM-2	IGG-2	
15:28:40	1.152		1.117	0.57	
				<Test	
Ser/Pl	N	00028-1			6
21/10/15	IGA-2		IGM-2	IGG-2	
15:28:58	0.970		0.859	0.49	
				<Test	
Ser/Pl	N	00028-2			7
21/10/15	IGA-2		IGM-2	IGG-2	
15:28:58	1.373		1.182	0.62	
				<Test	
Ser/Pl	N	00028-3			8
21/10/15	IGA-2		IGM-2	IGG-2	
15:28:58	0.823		0.640	0.29	
				<Test	
Ser/Pl	N	00028-4			9
21/10/15	IGA-2		IGM-2	IGG-2	
15:28:58	1.206		1.377	0.75	
				<Test	
Ser/Pl	N	00028-5			10
21/10/15	IGA-2		IGM-2	IGG-2	
15:28:58	0.951		0.580	0.29	
				<Test	
Ser/Pl	N	00029-1			11
21/10/15	IGA-2		IGM-2	IGG-2	
15:29:52	1.098		0.916	0.48	
				<Test	
Ser/Pl	N	00029-2			12
21/10/15	IGA-2		IGM-2	IGG-2	
15:29:52	1.023		0.804	0.43	
				<Test	

Ser/Pl	N	21/10/15	15:29:52	00029-3	IGA-2 0.978	IGM-2 0.970	13	IGG-2 0.52	<Test
Ser/Pl	N	21/10/15	15:29:52	00029-4	IGA-2 1.012	IGM-2 1.570	14	IGG-2 0.80	<Test
Ser/Pl	N	21/10/15	15:29:52	00029-5	IGA-2 0.791	IGM-2 1.007	CO 1	IGG-2 0.56	<Test
Ser/Pl	N	21/10/15	15:30:10	00043-1	IGA-2 0.704	IGM-2 1.355	Co 2	IGG-2 0.73	<Test
Ser/Pl	N	21/10/15	15:30:10	00043-2	IGA-2 0.593	IGM-2 0.588	Co 3	IGG-2 0.31	<Test
Ser/Pl	N	21/10/15	15:30:10	00043-3	IGA-2 1.383	IGM-2 2.457	Co 4	IGG-2 1.21	<Test
Ser/Pl	N	21/10/15	15:30:10	00043-4	IGA-2 0.621	IGM-2 0.331	Co 5	IGG-2 0.14	<Test
Ser/Pl	N	21/10/15	15:30:10	00043-5	IGA-2 0.660	IGM-2 0.964	Co 6	IGG-2 0.48	<Test
Ser/Pl	N	21/10/15	15:56:40	00023-1	IGA-2 1.409	IGM-2 1.303	3	IGG-2 0.71	<Test

Kontrollen 1–6 ↓

→ Probe 3 (Patientin)
